# 3D peripheral subtraction MRA using flow-spoiled ECG-triggered balanced SSFP

**DOI:** 10.1186/1532-429X-11-S1-P288

**Published:** 2009-01-28

**Authors:** Zhaoyang Fan, Xiaoming Bi, John Sheehan, James Carr, Jerecic Renate, Debiao Li

**Affiliations:** 1 Northwestern University, Chicago, IL USA; 2 Siemens Medical Solutions, Chicago, IL USA

**Keywords:** Peripheral Arterial Disease, Nephrogenic Systemic Fibrosis, Peripheral Arterial Disease Patient, Vessel Wall Imaging, Venous Contamination

## Introduction

Peripheral arterial disease (PAD) is a major cause of diminished functional capacity and quality of life in a large portion of western populations. While 3D contrast-enhanced (CE) MRA is becoming a modality of choice for clinical PAD examinations, the potential for nephrogenic systemic fibrosis (NSF) in patients with renal insufficiency has triggered a renaissance of interest in non-contrast enhanced (NCE) MRA. Various NCE-MRA strategies employing 3D half-Fourier FSE [1] or balanced SSFP (bSSFP) [2] have shown great promise. Recently, flow-sensitizing dephasing-prepared (FSD) bSSFP was proposed for vessel wall imaging [3]. The present work aimed to investigate the feasibility of MRA in lower legs utilizing FSD-bSSFP combined with ECG-triggering and image subtraction.

## Materials and methods

**Figure 1:**
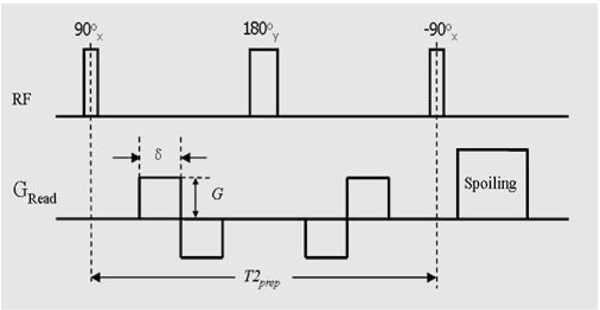
**FSD module diagram**.

The FSD module was modified by using bipolar gradient rather than unipolar gradient before and after the center 180-RF pulse to address the artifactual issue resulted from imperfect frequency response (Figure 1). Nine healthy subjects (5 M 4 F) were imaged at 1.5 T (Avanto, Siemens) using a peripheral phased-array coil and spine coils. Phase-contrast flow scan was first performed right above the popliteal trifurcation to derive the arterial flow peak time T. bFFSP scans were then conducted: (1) in 2 subjects, non-FSD-bSSFP (bright-blood, BB) scans and FSD-bSSFP (dark-blood, DB) scans (FSD gradient strength G = 10 mT/m and duration d = 1.2 ms, applied on x-axis) with 5 ECG-trigger delays (0, 1/2 T, T, 3/2 T, 2 T), and arterial blood SNR for each leg was measured from 8 ROI's; (2) in 9 subjects, BB scans triggered at mid-diastole (~2 T) paired with DB scans triggered at ~T using G = 5, 10,..., 25 mT/m (d = 1.2 ms), and MIP's were created from subtraction data sets and reviewed by an radiologist, using a four-grade scale (1, poor; 2, fair; 3, good; 4, excellent). bSSFP imaging parameters: TE/TR = 1.9/3.8 ms, centric ordering, 3 shots/partition, FOV = 400 × 311 × 67 mm3, matrix = 432 × 336 × 72, spectral fat sat, BW = 965 Hz/pixel, GRAPPA factor = 2, TA = ~3 min/scan.

## Results

**Figure 2:**
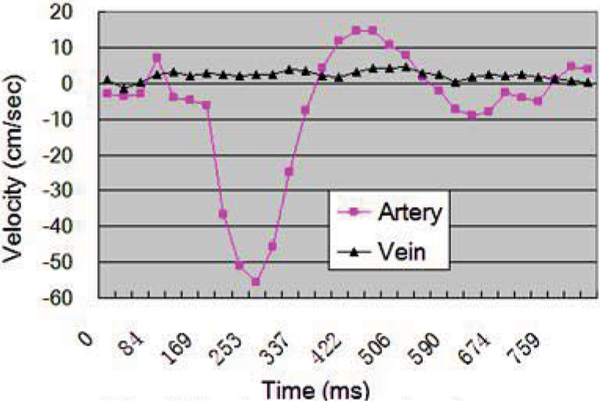
**Typical blood velocity curve in one cardiac cycle**.

**Figure 3:**
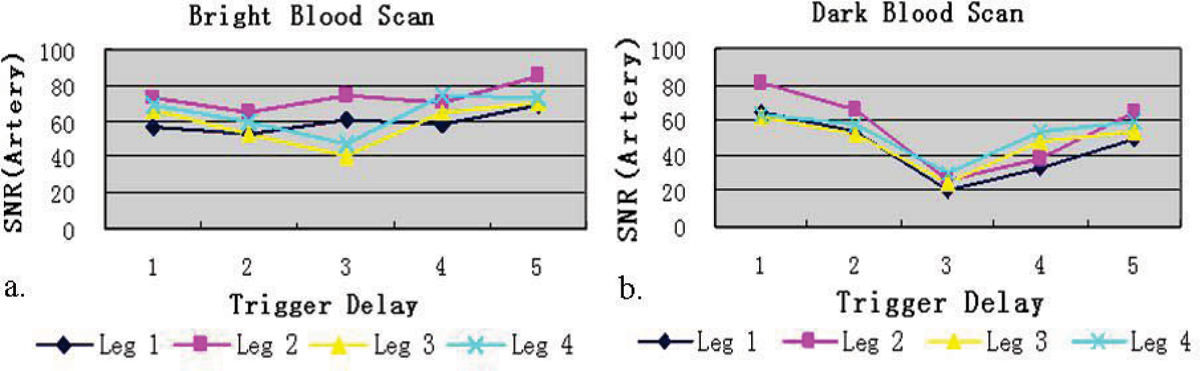
**Arterial blood SNR measured from 4 legs in BB (a) and DB scans**.

**Figure 4:**
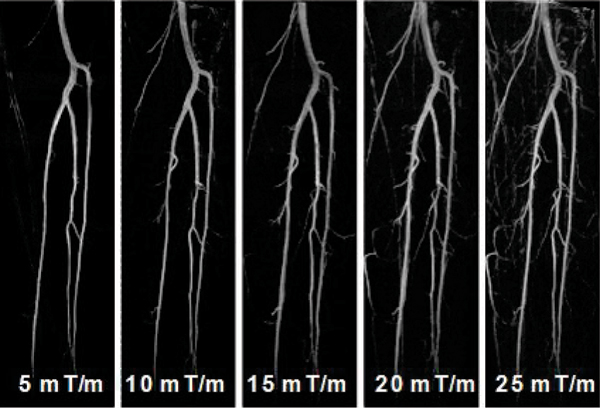
**MIP images after subtraction**. AS the FSD gradient increased, more arteries were shown, but venous signal might have interfered with artery visualization.

Average velocity within the lumen was considerably higher in the arteries than in the accompanying veins (Figure 2). In BB scans, higher arterial-blood SNR was achieved during diastole (Figure 3a), whereas DB scans showed superior flow void during systole (Figure 3b). On both scans, venous blood signal was barely affected. When FSD strength stepped from 5 to 25 mT/m, the arterial signal on the subtraction images were generally improved but venous contamination became problematic (Figure 4). In 18 legs, the counts of score 4 for G = 5, 10,..., 25 mT/m are 10(56%), 11(61%), 7(39%), 0(0%), 2(11%), respectively.

## Discussion and conclusion

In the case of a laminar flow, the faster average velocity and/or greater first gradient moment m1 conveyed by FSD, the higher likelihood the flowing spins are dephased and thus suppressed. Hence, the arterial blood is more susceptible to FSD with a weak m1 compared with venous blood during systole. Since bSSFP is not truly flow-compensated, however, bright blood scan can achieve substantially high arterial blood signal during mid-diastole. For those reasons, ECG-triggering facilitates subtraction MRA. The results indicate that FSD gradient strength, or more accurately m1, should be controlled to a low level (for lower legs here, G = 10 mT/m, m1 = 34.8 mTms2/m) to selectively suppress arterial blood and to avoid otherwise venous contamination. The feasibility of this approach was demonstrated on healthy distal lower extremities. Further investigation on PAD patients, with CE-MRA or x-ray angiography correlation, is warranted. It is anticipated that this strategy could be applied to other vascular territories where appropriate choice of m1 (magnitude and direction) would vary with the specific flow patterns.
